# Ecological Risk Evaluation and Removal of Emerging Pollutants in Urban Wastewater by a Hollow Fiber Forward Osmosis Membrane

**DOI:** 10.3390/membranes12030293

**Published:** 2022-03-04

**Authors:** Mónica Salamanca, Rebeca López-Serna, Laura Palacio, Antonio Hernandez, Pedro Prádanos, Mar Peña

**Affiliations:** 1 Institute of Sustainable Processes (ISP), University of Valladolid, Dr. Mergelina s/n, E-47011 Valladolid, Spain; monica.salamanca@uva.es (M.S.); rebeca.lopez@uva.es (R.L.-S.); antonio.hernandez@uva.es (A.H.); ppradanos@uva.es (P.P.); pena@iq.uva.es (M.P.); 2 Department of Chemical Engineering and Environmental Technology, University of Valladolid, Dr. Mergelina s/n, E-47011 Valladolid, Spain; 3 Department of Analytical Chemistry, Faculty of Sciences, University of Valladolid, Paseo Belén 7, E-47011 Valladolid, Spain; 4 Department of Applied Physics, Faculty of Sciences, University of Valladolid, Paseo Belén 7, E-47011 Valladolid, Spain

**Keywords:** urban wastewater, forward osmosis (FO), organic matter concentration, Contaminants of Emerging Concern (CECs), ecological risk quotient

## Abstract

Forward osmosis (FO) is a promising technology for the treatment of urban wastewater. FO can produce high-quality effluents and preconcentrate urban wastewater for subsequent anaerobic treatment. This membrane technology makes it possible to eliminate the pollutants present in urban wastewater, which can cause adverse effects in the ecosystem even at low concentrations. In this study, a 0.6 m^2^ hollow fiber aquaporin forward osmosis membrane was used for the treatment of urban wastewater from the Valladolid wastewater treatment plant (WWTP). A total of 51 Contaminants of Emerging Concern (CECs) were investigated, of which 18 were found in the target urban wastewater. They were quantified, and their ecotoxicological risk impact was evaluated. Different salts with different concentrations were tested as draw solutions to evaluate the membrane performances when working with pretreated urban wastewater. NaCl was found to be the most appropriate salt since it leads to higher permeate fluxes and lower reverse saline fluxes. The membrane can eliminate or significantly reduce the pollutants present in the studied urban wastewater, producing water without ecotoxicological risk or essentially free of pollutants. In all cases, good recovery was achieved, which increased with molecular weight, although chemical and electrostatic interactions also played a role.

## 1. Introduction

The development of new technologies for urban wastewater treatment deserves more and more attention due to the growing environmental restrictions at a global level and the increasing need to adapt conventional plants to more robust and sustainable treatment systems, necessarily combining energy efficiency and low environmental impact [1].

Currently, aerobic biological processes are the most widely used techniques for urban wastewater treatment. These procedures require a large amount of energy due to their high electricity demand, mainly for aeration, and produce excessive amounts of sewage sludge with the consequent environmental problem that this implies [2], consequently increasing the cost of the treatment. Furthermore, in these aerobically activated sludge processes, not all the energy content present in the wastewater is utilized, since the carbon content (i.e., chemical energy) of the wastewater is converted into biomass and carbon dioxide.

Alternatively, anaerobic treatment processes, such as those used for industrial wastewater, have recognized advantages over aerobic available treatments: lower energy consumption, less sludge production, energy generation in the form of methane, and the transformation of phosphorus into a chemical state more convenient for subsequent recovery [3].

The concentration of organic matter in urban wastewater is usually low, with a high proportion of particulate matter [4,5]. Therefore, a sufficient organic loading rate cannot be maintained in the anaerobic treatment of urban wastewater at low temperatures, resulting in low biogas yield and the inadequate removal of organic pollutants from wastewater. This low organic loading and the economic unfeasibility of treating such large volumes of wastewater have made anaerobic treatment for urban wastewater unattractive over the years. However, these limitations could be overcome by operating with previously concentrated urban wastewater to achieve a higher organic load so that operation under mesophilic anaerobic conditions would be made feasible [3,6,7].

Promising results concerning the up-concentration of urban wastewater by forward osmosis (FO) membrane processes can be found in the recent literature. In effect, it seems to have been proven that FO could make it possible to separate and concentrate the organic matter present in urban wastewater [8,9,10,11,12,13,14,15]. Thus, FO seems to be a promising technology for the preconcentration of wastewater [16,17,18].

Unlike pressure-driven membrane processes, the driving force in forward osmosis is the osmotic pressure gradient between the feed solution (FS) (wastewater) and the draw solution (DS). This implies an important advantage due to its lower energy consumption [19], and especially attending to the lower membrane fouling [15,20] that is lower than with ultrafiltration (UF) membranes which, in this case, increases due to the relatively high-pressure gradients needed in UF. The nature of the driving force in FO implies not only that the membrane has a lower susceptibility to fouling but also that the slight fouling that appears can be highly reversible [21]. Therefore, FO certainly has high potential to be used to concentrate the organic matter and nutrients in urban wastewater to a small volume in order to integrate it with anaerobic treatment to facilitate resource recovery.

Osmosis is the net diffusive transport of water through a selectively permeable membrane from a solution of low-solute concentration (low-osmotic pressure) to a solution of high-solute concentration (high-osmotic pressure). One key component for the successful development of FO technologies is the selection of the draw solution. One of the criteria for choosing the extraction solution is that it must have a higher osmotic pressure than the feed solution to produce a high flow of water permeate. [22]. Another criterion to take into account in the selection of the extraction solution is that the diffusive transport of salt should be minimal, that is, that the reverse flow of salt from the DS to the FS should be the lowest possible [23]. Therefore, it is important to select the correct type of salt and the right type of membrane, since if the reverse flow of salt is very high, it could pose a problem for the subsequent anaerobic treatment.

There are different types of draw solution that have been studied in forward osmosis processes with different types of membranes and for different applications. The effects of the different draw solutions depend on the configuration, type, and material of the membrane used in each case [24,25,26,27,28,29].

This type of process could be applied in urban wastewater treatment in coastal areas where the use of seawater as a concentrated salt solution makes its recovery unnecessary [30]. In the case of a lack of a natural saline water source, the procedure requires the system to be coupled to a reverse osmosis (RO) process to recover the DS solution. In this case, energy savings are reduced, but membrane fouling is still considerably reduced, increasing the useful life of the membranes and obtaining a stream previously diluted to some extent, making it easy to operate RO to obtain high-quality water for urban, agricultural, or industrial use [31].

On the other hand, the use of forward osmosis membranes compared to conventionally activated sludge treatment processes allows one to obtain a very high percentage rate of the rejection of CECs present in urban wastewater [32,33]. This is a serious issue as far as numerous studies to date have shown that a growing list of CECs from domestic, industrial, and agricultural practices put the natural environment and public health at risk [34]. Moreover, it has been concluded that the elimination of CECs from WWTPs is still insufficient and can be found in concentrations of ng/L to mg/L both in effluents and influents [35,36,37,38]. There are other possible methods, such as photocatalysis, that can be used to eliminate the toxicity and contaminants present in urban wastewater; this is an environmentally friendly option. However, this alternative is not the most suitable when it comes to urban wastewater [39]. Therefore, concentrating and being able to remove these contaminants from water is an excellent advantage of FO.

There are various membrane configurations, such as plate and frame modules, spiral bound, or hollow fiber. However, for applications that require small footprint designs as well as high volume gaps, hollow fiber (HF) modules can be advantageous. There are commercial hollow fiber modules that integrate aquaporin proteins that provide high water transport in a very selective way with high chemical resistance [40]. In addition, these hollow fiber membrane modules present a high rejection rate in terms of contaminants, as we saw in a previous study [41].

Considering the above background, the objective of this work was to study the behavior of a forward osmosis hollow fiber membrane in treating urban wastewater subjected to different pretreatments (centrifuged and filtered, only centrifuged, and without pretreatment). In addition, changes in the membrane flux, different types of salt in the DS, and different DS concentrations were investigated to determine the permeate flow and the reverse saline flow under each one of these conditions. The adsorption of organic matter on the membrane and/or in the system and its recovery after performing several osmotic washes was studied.

On the other hand, the presence of 51 emerging pollutants in urban wastewater at the outlet of the desander of the Valladolid WWTP was studied, as well as the concentration and recovery of these emergent contaminants when passing them through a forward osmosis hollow fiber membrane. Ecotoxicological risk assessment of these contaminants was also evaluated.

## 2. Materials and Methods

### 2.1. Forward Osmosis Membrane and Experimental Setup

**Figure 1:**
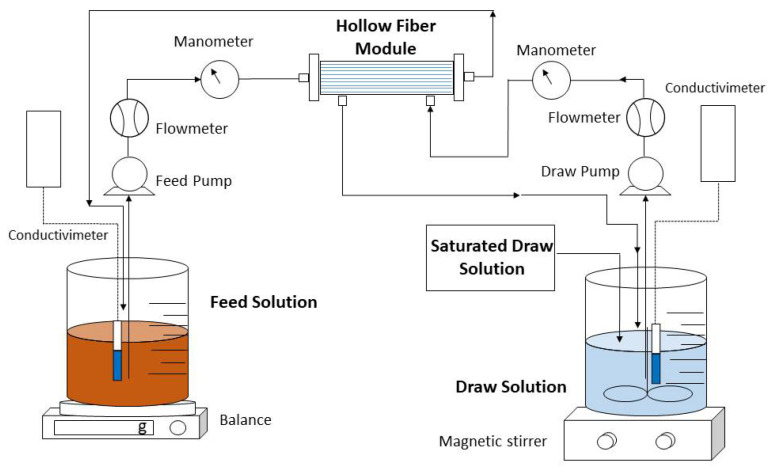
Experimental setup of concentrating urban wastewater by FO membrane.

An Aquaporin Inside^TM^ FO hollow fiber module (Aquaporin A/S, Kongens-Lingby, Denmark) was used throughout this study. The hollow fiber module contains 0.6 m^2^ of active surface area and consists of an active layer of thin-film composite polyamide (TFC) with integrated aquaporin proteins. Figure 1 illustrates the FO concentration system comprising a forward osmosis membrane module together with FS and DS reservoirs.

Closed countercurrent recirculation circuits of the feed and extraction solutions were carried out on each side of the FO membrane through two peristaltic pumps. In all experiments, the FS was passed through the lumen side of the hollow fibers (active side), while the DS was passed along the shell side. To calculate the flow of water that crosses from feed to draw and to observe the reduction in volume over time in the experiments, a digital electronic balance was used. In addition, a conductivity meter was inserted into the FS and DS to measure concentration and to evaluate the saline flux. To determine the FS and DS flow rates and the inlet and outlet pressures, a flowmeter and a manometer were placed on each side of the membrane. In all experiments, a saturated solution of draw solution was added and magnetically stirred to keep constant the concentration of the draw solution.

The list of Aquaporin Inside^TM^ FO hollow fiber module specifications provided by the membrane manufacturer is shown in Table S1 of the Supplementary Materials.

### 2.2. Urban Wastewater Pretreatment

In this study, urban wastewater from the wastewater treatment plant (WWTP) of the city of Valladolid (Spain) was used. Wastewater was collected at the outlet of the desander. The general characteristics of the collected urban wastewater as measured were an average of 345.6 mgO_2_/L in chemical oxygen demand (COD), 0.94 g/Kg in total solids (TS) and 0.41 g /Kg in volatile solids (VS). Samples were collected in drums and stored under refrigeration until use.

Different wastewater pretreatments were used in order to determine the behavior of the Aquaporin Inside^TM^ FO hollow fiber module. For that purpose, a Thermo Scientific Sorvall^®^ Legend^®^ RT Plus centrifuge (Waltham, MA, USA), a filter with a 0.7 µm pore size, and hydrophilic fiberglass with a diameter of 47 mm were used for the different pretreatments to which wastewater was subjected. Regarding the operating conditions of the membrane, a liter of NaCl was initially used as the draw solution with a constant concentration of 0.5 M; throughout the experiments, the FS flow rate of 0.55 L/min and a flow rate of 0.35 L/min for the DS were set, in accordance with the conditions selected in a previous work [41]. The duration of each experiment was 40 min, until the volume of the FS decreased by at least 60%, starting from a volume of urban wastewater of 3 L at the beginning of the experiment. Different wastewater pretreatments were used in order to determine the behavior of the forward osmosis membrane according to the following scenarios.

- Scenario A involved wastewater centrifuged at 10,000 rpm (revolutions per minute) for ten minutes and then filtered through a filter with 0.7 μm pores. The final characteristics of the urban wastewater obtained after this pretreatment and used as the FS for the FO process were 82.42 mgO_2_/L in COD, 0.89 g/Kg in TS, and 0.35 g/Kg in VS.
- Scenario B involved centrifuging wastewater at 10,000 rpm for ten minutes without filtering it. The final characteristics of the urban wastewater with this pretreatment, which were used as the FS for the FO process, were 146.15 mgO_2_/L in COD, 0.79 g/Kg in TS, and 0.36 g/Kg in VS.
- Scenario C involved using wastewater directly as the FS in an FO procedure without carrying out any pretreatment. The characteristics of this FS were those mentioned at the beginning of this section.

For all FO procedures in each scenario, data on the change in weight and conductivity measurements were taken every minute to later be used to calculate the reverse salt flow, $J_{s}$, and the water flow, $J_{w}$. The DS concentration was kept constant by the controlled addition of a saturated NaCl solution.

For the rest of the experiments, urban wastewater with pretreatment A was used as the FS, since the manufacturer and some authors recommend a filtering pretreatment prior to the use of the membrane to minimize fouling and increase permeate fluxes [13,42,43].

After each test, the membrane was washed with Milli-Q^®^ water (Burlington, MA, USA) in both solutions until a conductivity of around 10 µS/cm was obtained. In the case of pretreatment C, it was necessary to open the module to remove the dirt accumulated at the entrance of the fibers. At the end, a control test (milli-Q water as the FS and NaCl 0.5 M as the DS) was performed to ensure that there was no significant fouling due to the pretreatments used.

### 2.3. Experiments with Different DS Chemicals and with Different Concentrations of NaCl

Other chemicals were studied to determine the effect of different DSs in the flow in order to improve the efficiency of the process. To do this, sodium chloride (NaCl), magnesium sulfate heptahydrate (MgSO_4_·7H_2_O), glucose (C_6_H_12_O_6_), sodium acetate (CH_3_COONa), and magnesium chloride hexahydrate (MgCl_2_·6H_2_O) were used. The experiments lasted 20 min; the volume of urban wastewater with pretreatment A as the FS used was 2.5 L, and the volume and concentration used for each DS was 0.5 L and 0.5 M, respectively. The concentration of the draw solutions was kept constant by adding saturated solutions of each DS as needed. In all cases, a conductivity meter Hanna mod. HI 5522 (Woonsocket, RI, USA) was used to monitor the conductivity of the draw solutions, and a magnetic stirrer was used to ensure homogeneity. However, in the case of glucose, a Abbe-2wa refractometer (PCE-Iberica, Albacete, Spain) was used instead of a conductivity meter in order to control and keep the concentration of the solution constant. The change in weight of the FS and the conductivity (or refraction index) in the draw and feed solutions were collected every minute throughout the duration of each experiment. This was to calculate the water and reverse fluxes.

Experiments have also been carried out with different concentrations of NaCl to find which could be a more optimal concentration in urban wastewater. The concentrations tested were 0.5 M, 1.0 M, 1.5 M, and 2.0 M with NaCl as the DS, and the run time of the experiments ranged from 36 min to 62 min.

At the end of every experiment, the membrane was washed using milli-Q water until a conductivity below 10 μs/cm was reached in both the feed and draw solution vessels. In the same way as in Section 2.2, after each cleaning process, a control test of the membrane module was carried out to check that it maintained its initial properties. All measurements were carried out at a temperature of 298 K and with the same flow conditions.

### 2.4. Emerging Contaminants in Urban Wastewater

Fifty-one pollutants from the WWTP of the city of Valladolid were studied. The presence of these pollutants in urban wastewater was evaluated, and their concentration in these waters was quantified. In addition, the concentration of these contaminants after passing through the FO membrane was measured, and the membrane’s rejection capacity against each contaminant was evaluated. The total list of the 51 contaminants studied can be found in the Table 1.

The experiment was carried out by using urban wastewater with pretreatment A as the FS and 0.5 M of NaCl as the DS. The flow rates used are mentioned above in Section 2.2. All measurements were carried out at a pH of around 7. The operation consisted of feeding slightly more than 5 L of urban wastewater previously centrifuged and filtered, until a considerable reduction in the feed volume was achieved of about 87% in 62 min. Samples were collected from the FS at time 0 and from the FS at the end of the experiment and immediately stored in the freezer at −20 °C until analysis.

#### Ecological Risk Assessment of CECs

For the ecological risk assessment, an estimated risk ratio (RQ) was calculated for each CEC using the following Equation (1).

(1) $$\mathrm{Risk}\;\mathrm{Quotient}\;\left(\mathrm{RQ}\right)=\frac{C_{X\;\left(\mathrm{effluent}\right)}}{\mathrm{PNEC}}$$

with *C_X_* (effluent) being the concentrations in the final treated effluent (in ng L^−1^) and PNEC the predicted no-effect concentrations (in ng L^−1^) that up to now have not always been available in the literature. Therefore, PNECs are typically calculated from EC50 values (half maximal effective concentration: concentration in mol/L required to obtain a 50% of the maximal effect) corrected by a safety factor of 1000, as recommended by the Water Framework Directive [44]. RQ values less than 0.1 indicate a low risk, an RQ between 0.1 and 1.0 corresponds to a moderate risk, and an RQ greater than or equal to 1.0 means that there is a high risk [45,46].

### 2.5. Analytical Methods

Key parameters such as the COD, TS, and VS of urban wastewater from WWTP effluents were measured according to standard methods. Appropriate dilutions and adjustments were made to minimize chloride interference during COD measurements on the samples. A Shimadzu analyzer (TOC-L) was used to determine the TOC concentration in some of the samples.

The samples with the emerging contaminants were analyzed directly by Ultra-High-Performance Liquid Chromatography (UHPLC)–tandem Mass Spectrometry (MS/MS) in Selected Reaction Monitoring (SRM) mode by SCIEX Triple Quad™ 6500+ LC System-MS/MS, both from SCIEX (Framingham, Massachusetts, USA) [40]. The injection volume of the samples was 200 µL, and a matrix-matched calibration curve was necessary for the quantification of the analytes in the samples. The complete list of SRM parameters is provided in the Supplementary materials (Table S2), as well as the Method Limits of Detection (MLD) and Quantitation (MLQ) for each target analyte (Table S3), which was determined according to the lowest concentration from the calibration curve which gave a signal-to-noise ratio (S/N) larger than 3.0 and 10.0, respectively.

## 3. Results

### 3.1. Effect of Different Urban Wastewater Pretreatments

The pretreatment urban wastewater used as the FS has a direct effect on the efficiency of the FO procedure. The pretreatment affects the water flux, the reverse flux of solutes, and fouling of the membrane. To fully understand the effects of wastewater pretreatment on FO, three scenarios were observed, as mentioned in Section 2.2. These were pretreating wastewater by:

A—Centrifuging at 10,000 rpm and filtering by 0.7 μm;

B—Centrifuging at 10,000 rpm; 

C—Urban wastewater without any pretreatment.

**Figure 2:**
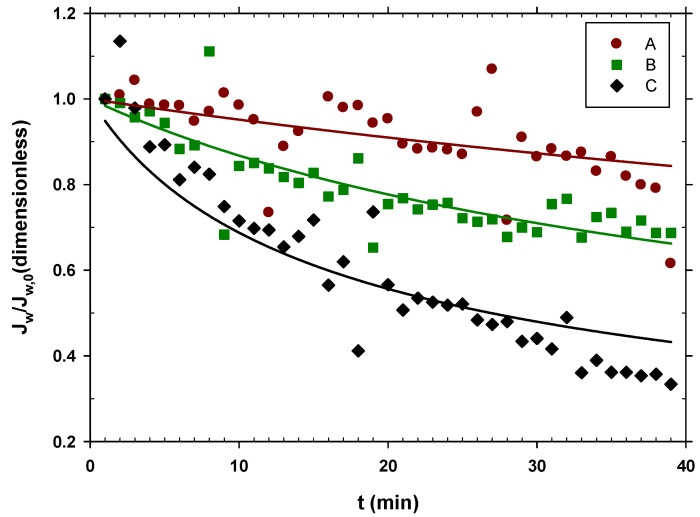
$J_{w}/J_{w,0}$ in time with different pretreatments of urban wastewater. A, centrifuged at 10,000 rpm and filtered by a filter with 0.7 μm pores; B, centrifuged at 10,000 rpm; C, urban wastewater without any pretreatment.

The corresponding water fluxes relative to the initial ones, $J_{w}/J_{w,0}$, are shown in Figure 2 versus time.

To calculate $J_{w}$, the permeate water flux, Equation (2) was used.

(2) $$J_{w}=\frac{V_{FSt_{i+1}}-V_{FSt_{i}}}{A\left(t_{i+1}-t_{i}\right)}$$

where $V_{FSt_{i+1}}$ and $V_{FSt_{i}}$ are the feed volumes in times $t_{i+1}$ and $t_{i}$, respectively, and A is the surface area of the active side of the membrane, which is constant.

The drop in flow has been attributed to the deposition of substances present in urban wastewater (WW), including molecules and particles, on the membrane surface. However, it is also possible that increased viscosity or plugging of some fibers due to the presence of big particles may have an influence.

Figure 2 shows that experiment A exhibited almost constant water flow throughout the experiment and had the highest one among the three scenarios. In this case, the used wastewater had a lower content of organic matter, and therefore, there was less fouling, and the water flow decreased only slightly. This shows that centrifuging urban wastewater at 10,000 rpm and then filtering it by 0.7 μm helps to keep a constant and relatively high water flow, as Figure 2 shows.

Experiment B with only centrifuged water showed how the flow decayed more easily than in case A, showing that the microfiltration through the 0.7 μm filter still gave relatively clean water, and therefore, high flows.

Experiment C revealed how the use of urban wastewater significantly reduced the flow of water from the FO membrane over time.

A Cake type fouling model can be assumed, since most of the substances present were much larger than the pore size, and as the process progressed, the concentration of the retained substances increased. In accordance with this hypothesis, the flow values, $J_{w}$, were adjusted in relation to the initial flow of each scenario, $J_{w,0}$ with the following formula [47]:

(3) $$\frac{J_{w}}{J_{w,0}}={\left[1+Kt\right]}^{-1/2}$$

where *t* is the elapsed time and K is the kinetic constant of the fouling process. The curves in Figure 2 are those that correspond to their fitting to Equation (3) with the resulting parameters shown in Table 2. As expected, the fouling constant K increases as the organic matter content of the WW increases.

As observed in Table 2, the average water flux for scenario A was quite high; in fact, it only showed a reduction of 4.6%, as compared to similar experiments with milli-Q water; in this case, $J_{w}$ was 8.4 LMH (L/m^2^h) [41]. This means that in this case, the membrane practically did not get dirty. For case C, a reduction of 41% was found, which is reasonable when we consider that totally raw wastewater, without any pretreatment, was used.

For the case of the reverse flow of salt, in the previous study, with milli-Q water, $J_{s}$ was 0.65 GMH (g/m^2^h). Now, for the case A, the reduction was 3%, which is also quite similar to that obtained for milli-Q water. In case C, there was an increase of 15% for the reverse flow. This increase was due to the fact that $J_{s}$ is proportional to the concentration gradient between both sides of the membrane, and a lower $J_{w}$ concentrates NaCl in the DS side, and therefore, the concentration gradient is greater, increasing $J_{s}$. Obviously, when considering ionic transport, the charge on the membrane itself plays a relevant role. This charge varies with the nature of the solution in contact with it and the history of the membrane itself. These two factors may explain the fact that in case B, a value of $J_{s}$ a little lower than what could be expected was obtained.

**Figure 3:**
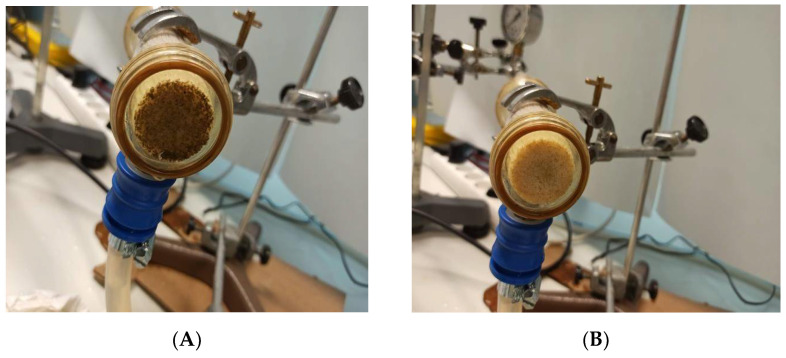
Scenario C. (**A**) Lumen plugging after use. (**B**) Membrane after opening and physical cleaning.

As can be seen in Figure 3, strong fouling appeared for case C, eventually leading to clogging of the fiber lumens, blocking the entrances to the hollow fibers that make up the membrane.

Considering the results obtained and the manufacturer’s recommendations for this type of membrane, it was decided to use pretreatment A for the rest of the experiments, since fouling was lower and higher fluxes were reached.

From time to time, after experiments with urban wastewater, quality control of the membrane was performed with milli-Q water to determine if the initial conditions of the membrane were maintained. It was observed that both the flow rate, $J_{w}$ (6.26–7.55 LMH), and the reverse flow rate of salt, $J_{s}$ (0.64–1.25 GMH), only changed slightly. These quite small changes may be due to both fouling and aging of the membrane.

The high level of fouling of the membrane when no previous pretreatment is used suggests that sedimentation, filtration with sand beds, and/or coagulation–flocculation treatment should be effective in reducing fouling of the membrane to scale up the procedure. However, on a smaller scale, pretreatment by centrifugation was chosen due to its rapidity and easy usage with small amounts of wastewater. In any case, filtering should be necessary to avoid any significant plugging of the hollow fibers at their inlet. With this type of pretreatment, it would be possible to concentrate the organic matter so as to perform anaerobic treatment.

### 3.2. Effect of Different DS

FO is a technique that is not limited by the type of extraction solution used, as indicated in the literature [22,24,28]. It is for this reason that five different types of draw solutions were tested, and their results compared. Inorganic salts 1:1 (sodium chloride (NaCl)), 2:1 (magnesium chloride hexahydrate (MgCl_2_·6H_2_O)), 2:2 (magnesium sulfate heptahydrate (MgSO_4_·7H_2_O)), and charged (sodium acetate (CH_3_COONa)) vs. neutral (glucose (C_6_H_12_O_6_)) organic species were selected as mentioned. 

**Figure 4:**
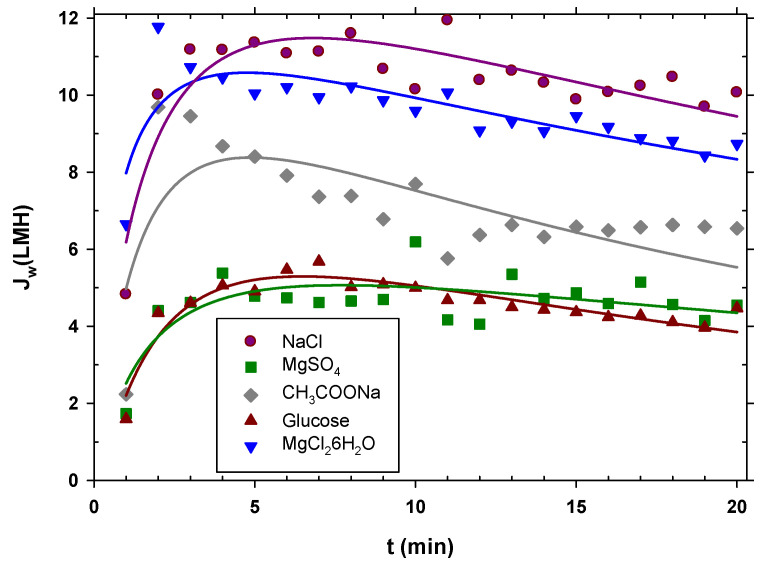
Water flux for the different types of draw solutions used over time. The curve is just a visual guide.

The experiments were carried out starting from urban wastewater with pretreatment A; the extraction solution was kept constant at 0.5 M, and the duration of each experiment was 20 min. It is observed in Figure 4 that sodium chloride showed the highest permeate flux, then magnesium chloride, then sodium acetate, and finally, with very similar water fluxes, magnesium sulfate and glucose. The initial flux at the beginning was lower than afterwards because the system had not yet reached equilibrium. In effect, the peristaltic pumps had to initially eliminate air bubbles until the established flow rate was reached and, therefore, the flow rate was lower than it should have been. Then, the flow decreased due to fouling; since the concentration of DS throughout the experiment remained constant, it can be inferred that the decrease in the flux was due to membrane fouling. Considering the aforementioned aspects, the curved lines in Figure 4 are no more than simple visual guides to the flows of each type of salt.

**Figure 5:**
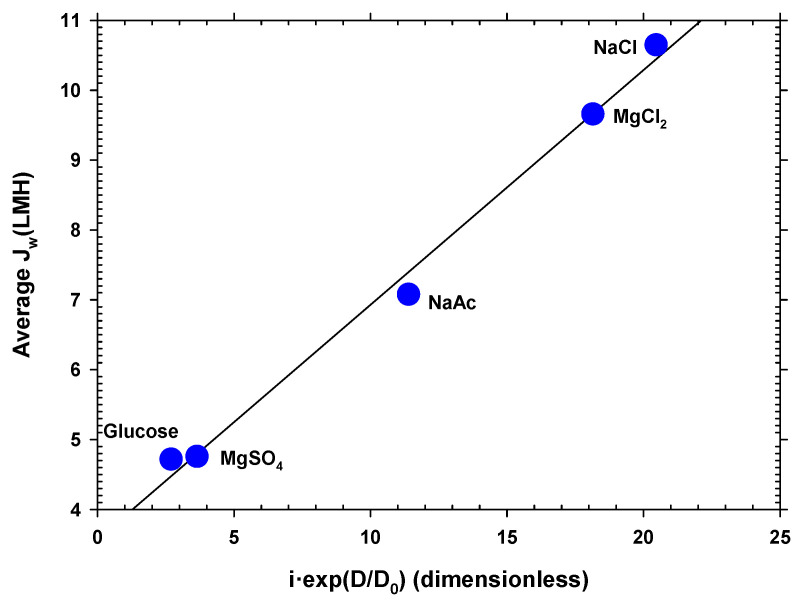
Average water flux versus a correlation between the Van’t Hoff factor and the diffusion coefficient of the different solutes. Here, NaAc stands for sodium acetate (CH_3_COONa).

Water flow is mainly a function of the osmotic pressure difference between both faces of the membrane. The osmotic pressure depends on the concentration of ions or neutral molecules present on both sides of the membrane. Since the molar concentration in the DS side studied was the same for all the substances, the osmotic pressure difference mostly depended on the Van’t Hoff factor, *i,* of each solute. In addition, the concentration was also influenced by the diffusion coefficient, because this caused the concentration gradient inside the porous layer on the DS side (caused by $J_{w}$) to decrease when the diffusion coefficient (*D*) was large. In fact, according to film theory, this difference in concentrations depends, among other factors, on the exponential of the diffusion coefficient. Taking these premises into account, a linear relationship was found between the mean water flow ($J_{w}$) for each solute and the product of the Van’t Hoff factor and the exponential of the diffusion coefficient shown in Equation (4):

(4) $$J_{w}=\alpha \;i\;e^{\left(D/D_{o}\right)}$$

where *D_o_* is the diffusion coefficient of the least diffusive solute and *α* is a constant. In Figure 5, it is clearly appreciated that the most appropriate solute is the most diffusive, with factor *i* close to 2. The values of the Van’t Hoff factors and the diffusion coefficients used for Figure 5 are shown in Table S4 of the Supplementary Materials [48,49,50,51]. Magnesium chloride, despite having a value of *i* close to 3, produces less flux due to its lower diffusion coefficient. Oppositely, magnesium sulfate and glucose, with values of *i* close to 1 and lower diffusion coefficients, give the lowest fluxes. This correlation can be highly useful as a guideline when selecting a solute to optimize the osmotic driving force in an FO process.

To calculate $J_{S}$, the reversal salt flux, Equation (5), was used.

(5) $$J_{S}=\frac{C_{FSt_{i+1}}V_{FSt_{i+1}}-V_{FSt_{i}}V_{FSt_{i}}}{A\left(t_{i+1}-t_{i}\right)}$$

where $C_{FSt_{i+1}}$ is the salt concentration of the feed solution in time $t_{i+1}$, $C_{FSt_{i}}$ is the salt concentration of the feed in time $t_{i}$, $V_{FSt_{i+1}}$ and $V_{FSt_{i}}$ are the feed volumes in times $t_{i+1}$ and $t_{i}$, respectively, and A is the surface area of the active side of the membrane.

The reversal solute flux for glucose was not measured because the corresponding water flux was extremely low. It was observed that sodium chloride stood out in terms of reversal flux and by having the lowest value. The high water flux and low reversal flux for sodium chloride indicate that this compound is the best choice to be used as a DS. The high water flux when sodium chloride was used indicates its efficiency in recovering water from wastewater feed. Its low reversal flux indicates that its use does not alter the feed solution substantially and that fouling of the membrane is low. In addition, it must be taken into account that the saline permeability depends on the charge of the membrane and the ions as well as on the size of the ions and their diffusion coefficient within the pores.

It seems clear that sodium chloride is and was expected to be one of the draw solutions with the highest water flow, as has been pointed out in the literature [22,28]. 

**Figure 6:**
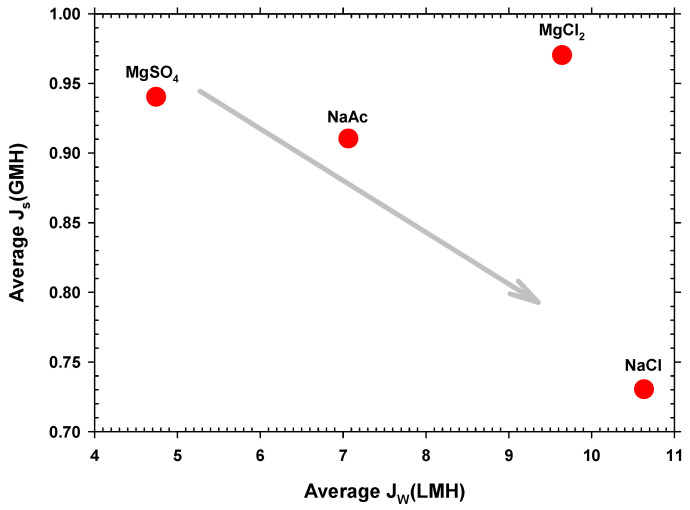
Average reverse salt flux versus each type of draw solution.

$J_{s}$ should increase with the saline permeability of the membrane and with the concentration gradient between the two faces of the active surface. At first, an increase in $J_{w}$ should decrease the concentration on the side of the active layer in contact with the DS (and therefore $J_{s}$), as observed in Figure 6 for all the studied salts except MgCl_2_. In this case, the concentration of Cl^−^ anions is twice that of the other salts, increasing the concentration gradient. Given that the membrane is negatively charged, it means that Mg^2+^, with two positive charges, could pass through the pores easier than Na^+^ [52]. Furthermore, Mg^2+^ is smaller than Na^+^, as shown in Table S5 in the Supplementary Materials [53]. All of these factors can explain the anomalous behavior of MgCl_2_ with quite high average reverse flux.

Hence, the sodium chloride DS experiments exhibited the highest permeate flux and a low reverse salt flux, which made them the most suitable as well as the most economical draw solutions to use. Nevertheless, although there is a low reversal flux, an accumulation of salinity in the FS could appear, which is a major limitation for high-retention membrane systems such as FO, particularly when it is combined with a biological process. The accumulation of salinity in FO systems can have detrimental effects on water flow, as the osmotic pressure of the feed solution increases, leading to a decrease in the osmotic driving force. Furthermore, the high salt content within preconcentrated wastewater could have adverse effects on subsequent anaerobic treatment processes [23]. A promising potential solution to mitigate salinity build-up, which is highly critical, in preconcentrated FO wastewater would involve the use of ionic organic extraction solutes. A possibility should be sodium acetate, which in our case gives fairly high water flow, although not so high as NaCl, with a reasonably low reverse salt flow thus mitigating the problem of salinity [12].

### 3.3. Effect of Different NaCl Concentration as Draw Solution

Once the sodium chloride solution was established as the most ideal from the list of tested solutes without considering the accumulation of salinity, different concentrations of sodium chloride were studied to observe the effect of concentration to concentrate organic matter in wastewater. The experiments were carried out using NaCl as the DS with concentrations of 0.5 M, 1 M, 1.5 M, and 2 M.

**Figure 7:**
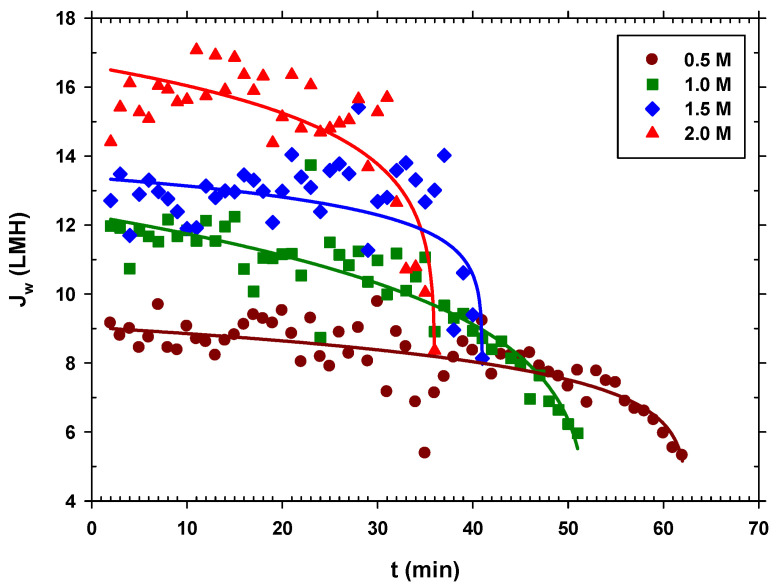
Water flux over time for each NaCl concentration (0.5 M, 1.0 M, 1.5 M, and 2.0 M). (Solid lines are just a visual guide to analyze trends).

In all the experiments, we started with approximately 5 L of centrifuged and filtered urban wastewater, and the system was operated until a very small volume of concentrated urban wastewater was left in the feed side (around 0.3–0.4 L). The concentration of the sodium chloride extraction solution was kept constant in each case. In Figure 7, we see that the higher the DS concentration, the greater the permeate flow, which seems logical since the osmotic pressure difference is greater, and the operating time is shorter. In addition, it is observed in all cases that during a large part of the process, the flow is almost constant, until there is a sudden drop in $J_{w}$ when the FS is highly concentrated, getting close to the DS concentration, thus decreasing the driving force. This appears to be associated with a critical fouling point, after which, tangential flow drag is no longer effective.

**Figure 8:**
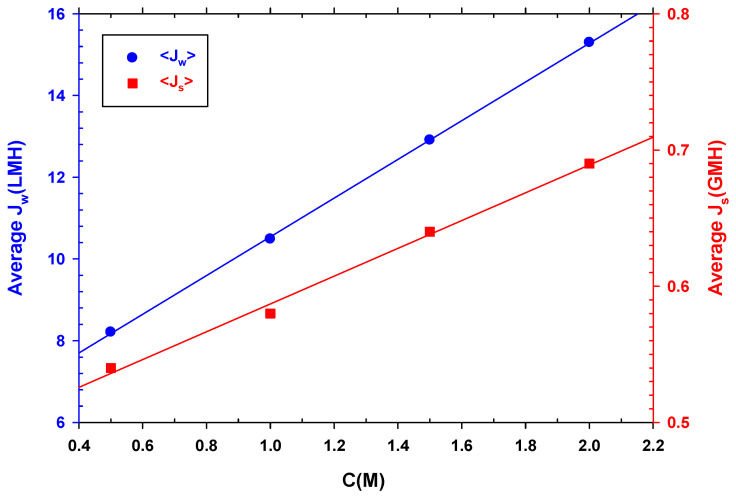
Average water flux (**left** side) and average reverse salt flux (**right** side) versus NaCl concentration.

As expected, and seen in Figure 8, both the average water and reverse salt fluxes increase linearly with DS concentration, since increasing concentration increases osmotic pressure that, in turn, increases water flux and simultaneously back diffusion of salt to the feed side. Therefore, although it would be ideal to use a DS with a higher concentration to increase the recovery of water from wastewater and concentrate it, reverse flow should also be considered, as it would be detrimental for the overall performance. 

If we compare $J_{w}$ and $J_{s}$, while $J_{w}$ almost doubles its value between 0.5 and 2 M, $J_{s}$ only increases by 28%. However, $J_{s}$ is proportional to the concentration gradient between the two membrane faces. This behavior is also associated with the dilution of the DS side which, as it increases with the salt concentration, causes $J_{w}$ to increase as well.

It must be taken into consideration that the reverse flow of chlorides from the DS to the concentrated FS during the experiments interfered with the measurement of the chemical oxygen demand (COD) of the concentrated feed solution. COD tests were necessary to determine the success of FO in concentrating wastewater. Moreover, high water fluxes caused by increasing DS concentrations are paired with a higher possibility of salinization of the feed and with a shorter time for the falling in water flux. Altogether, along with the increase in process costs incurred by increasing the salt concentration in the DS, this suggests that a concentration of 0.5 M of NaCl may be appropriate for the process of concentrating organic matter by FO. Another advantage of using 0.5 M is that in coastal areas, seawater with a similar concentration can be used as a draw solution [30,42].

### 3.4. Recovery of Organic Matter in the Forward Osmosis Process

Actually, no significant differences were found in the recovery of organic matter neither according to the type of salt nor according to the concentration of NaCl. In fact, amounts below 100% were recovered. Both FS and DS washing waters were analyzed to study if there was a high percentage retained in the membrane, if part of the organic matter was oxidized, or if it passed to the other side of the membrane and was picked up at the DS.

**Figure 9:**
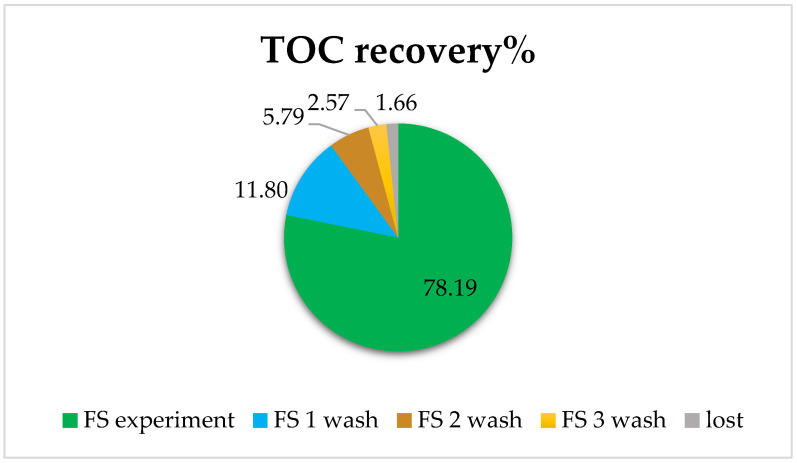
Total Organic Carbon (TOC) recovery in aquaporin forward osmosis membrane. FS corresponds to the %TOC after the experiment; FS wash corresponds to the %TOC after the membrane washes according to the order FS 1 wash, 2 wash, and 3 wash.

This was studied using osmotic washes of the pipes. In effect, three 15 min washes of water were performed. This was considered sufficient to recover the remnants that may have stayed within the membrane. For this, an experiment was carried out with centrifuged and filtered urban wastewater as the FS and a solution of 0.5M NaCl, and samples were taken from each side after 20 min. Subsequently, the three successive washes of 15 min were carried out, and samples of both FS and DS were collected to analyze the evolution of organic matter content. The Total Organic Carbon (TOC) recovery results in FS obtained by the TOC analyzer are shown in Figure 9.

It was observed that in the experiment with urban wastewater, 78% of TOC was recovered, and with three washes, practically everything that had been adsorbed on the membrane or in the system was recovered (only a remaining 1.66% loss of TOC appeared in this case).

The recovery of organic matter was also calculated by COD, where recoveries over 100% were evaluated, probably due to the interference of chlorides in its determination; the results are shown in Table S6 in the Supplementary Materials. In addition, in Table S7 of the Supplementary Materials, the recoveries for inorganic carbon (IC) and Total Carbon (TC) are shown. In these cases, the recovery found was lower than 100%. This may be because inorganic carbon is in the form of carbonates, and chemical rebalances cause some of it to pass into CO_2_ and be lost as a gas. Furthermore, a small part of carbonate can pass through the membrane, although this will not be a problem, because in the DS, it will contribute to the osmotic pressure of the system. 

### 3.5. Contaminants of Emerging of Concern (CECs)

#### 3.5.1. CECs in Urban Wastewater

Out of the 51 contaminants studied from different groups, 18 were found in urban WWTP from Valladolid in quantifiable concentrations, as seen in Table 3. The rest of the compounds were below the Method Limits of Detection (MLD), or the Method Limits of Quantification (MLQ), found in Table S3 of the Supplementary Materials.

It should be noted that the range of concentrations is of the same order at the entrance and exit of the WWTP, which seems reasonable, as WWTPs are not designed to eliminate this type of pollutant [54,55]. For this reason, there are many recent studies related to different alternatives to eliminate these types of pollutants [37,56,57,58,59,60,61]. 

Many of these pollutants have been found in waters from other WWTPs. Regarding the concentration found in those cases, it varies according to the country, daily amount consumed, time of year, collection time, and conservation, in addition to the experimental variability [36,38,62,63]. 

Most of the compounds that have been found in urban wastewater are substances of daily use by humans [64,65] such as drugs. Within this group are antibiotics (sulfamethoxazole, trimethoprim, sulfapyridine, ofloxacin, clarithromycin, levofloxacin, and ciprofloxacin) analgesics (naproxen, diclofenac, and ibuprofen) antihypertensives (atenolol), stimulants (caffeine), insect repellant (DEET), antiparasitics (fenbendazole), preservatives (methylparaben), and drugs in general for different diseases (gemfibrozil, atorvastatin, and carbamazepine). 

Molecular weights (MWs) as well as octanol–water distribution coefficients (K_ow_), which were used to determine hydrophobic/hydrophilic character [66,67] for the 18 contaminants that were found, were taken from the SciFinder database and can be found in the Table S8 of the Supplementary Materials.

For the compounds that could be quantified and are present in the urban WWTP, more specific characteristics were studied, such as the load of the compounds at a given pH as well as the log D. The characteristics of the pollutants present in urban wastewater can be found in Tables S9 and S10 in the Supplementary Materials.

#### 3.5.2. Ecological Risks and CECS in Wastewater

Numerous studies have used these concepts to assess the risk of contaminants in wastewater [36,68]. The ecological risk assessment (RQ) of pollutants has been studied to contextualize and analyze whether the concentrations obtained in urban wastewater in Valladolid can be considered dangerous for the three reference groups: daphnia, fish, and green algae. It makes sense that the organisms studied to assess the risk are aquatic, since natural waters are the first environmental compartment to receive effluents from the treatment plant. The three aquatic organisms studied (fish, green algae, and Daphnia magna) are standard species in ecotoxicity tests (recommended by organizations such as the CE, OECD, and ISO) and are presented as bioindicators to assess environmental risk. In addition, they belong to three different orders of the food chain; thus, they provide an idea on how the concentration of contaminant affects different levels of the aquatic food chain. For this, Equation (1) was used. In the Supplementary Materials, in Table S11, the EC50 values for each contaminant found in the literature are shown [69,70,71,72,73,74,75,76].

As seen in Table 4, a high risk (RQ > 1) is obtained for caffein and a medium risk (0.1 < RQ <1) for ibuprofen, atorvastatin, and fenbendazole for daphnia. For fish, there is a moderate risk of ibuprofen, atorvastatin, and caffeine. In the case of green algae, there is a high risk for sulfamethoxazole, ibuprofen and ciprofloxacin and a moderate risk for atorvastatin and atenolol. The rest of the pollutants not named presented a low risk (RQ < 0.1) for the three groups studied. Therefore, there are several pollutants that have a high risk, and this can affect the ecosystem. From Table 4, it can be seen that among the selected aquatic species, fish have shown relatively more resistance to the effect of contaminants.

Because CECs are not biodegradable and persist in the environment, they produce hazards that can negatively affect aquatic life and human health. Some of the negative effects that the presence of micropollutants in the environment can cause are toxic biological effects, such as estrogenicity, genotoxicity, and mutagenicity. Hormonal alterations and reproductive anomalies can be due to exposure to endocrine disruptors such as methylparaben [38]. Other possible effects are due to the inhibition of the growth rate of the organism, reduction in fertility, oxidative stress, reduction in steroid hormones, impacts on cardiovascular development, and neurotoxic effects; an example of these effects are exposure to diclofenac and carbamazepine [36,65].

#### 3.5.3. Concentration and Recovery of CECs

A concentration experiment was carried out to find out how much the contaminants in urban wastewater are concentrated and to see the recovery of these when they pass through a hollow fiber forward osmosis membrane once the experiment is finished. This may imply that a significant portion of the compounds tested may be retained inside the membrane fibers. Based on the previous results (see Figure 6), the process is more effective with NaCl in the DS, which is why this salt was chosen to be used in the study of the recovery of CECs. For this, the experiment began with approximately 5 L of centrifuged and filtered urban wastewater and a solution of 0.5 M of NaCl, and it was kept operating for 62 min, up to an 87% reduction in feed volume. During this process, some CECs may not have had time to reach adsorption equilibrium with the membrane material. However, we cannot ensure that the adsorption of the CECs can modify the recovery value for longer measurement times but, when this parameter was determined, the recovery values for longer times were shown to be similar or slightly higher than those presented in this study. The $J_{w}$ in the experiment was 7.63 LMH, and the $J_{s}$ was 0.62 GMH.

**Figure 10:**
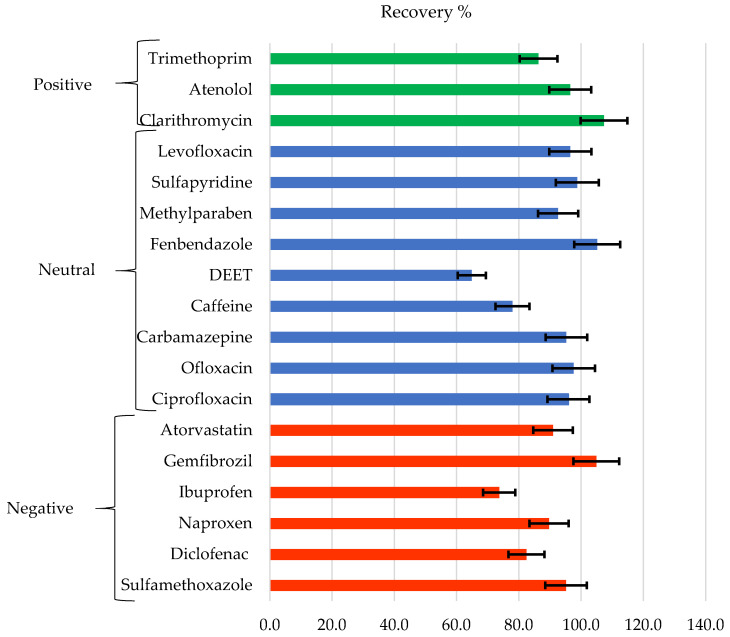
Recovery of contaminants grouped according to their charge at pH = 7.

Figure 10 shows the recovery of the analyzed chemicals classified according to their charge at pH 7 (positive, negative, and neutral), and error bars are attached to the recovery values with a 95% confidence interval. As can be seen, the recoveries obtained are higher than 80%, and many are very close to 100%, except for some cases such as DEET, caffeine, and ibuprofen. If the recovery data are compared with those previously obtained for some of these compounds, but dissolved in pure water [41], it is observed that in general, the compounds that display high recovery rates in pure water tend to provide high recovery rates in WW. No clear tendency is observed as a function of its charge, but a dependence in the recovery of the compound with the molecular weight is observed. Compounds with higher molecular weights such as atorvastatin, ofloxacin, ciprofloxacin, fenbendazole, levofloxacin, and clarithromycin obtain better recoveries than those with low molecular weights such as ibuprofen, caffeine, and DEET.

**Figure 11:**
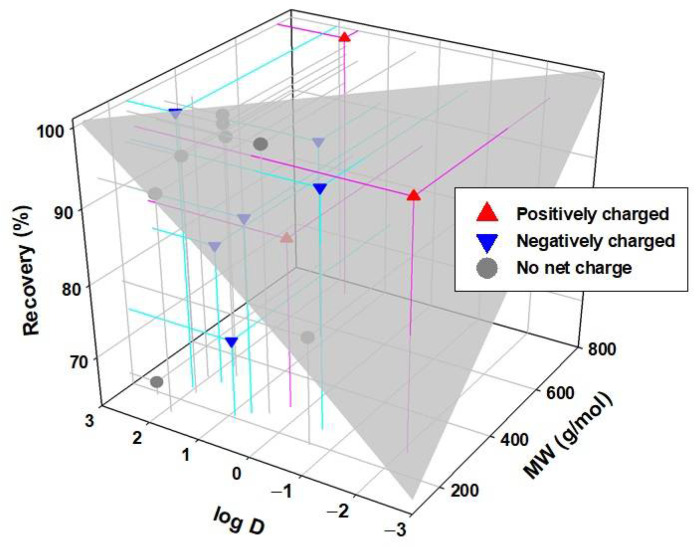
Recovery in the FS of the different CECs after the FO processes against the molecular weight and log D.

It is generally appreciated that the increase in MW and hydrophobicity increase recovery. However, some compounds do not align with this trend. As seen in Figure 11, atenolol that has a low MW, which means it would be expected to have low recovery. Actually, it shows a high adsorption capacity when faced with negatively charged surfaces due to its positive charge [41]. It would, in this case, also have a high affinity for some of the compounds present in WW, possibly due to the presence of negatively charged particles. A high adsorption or affinity process with some other compound present could also explain a greater than expected retention for sulfamethoxazole. In addition, there may be multiple chemical and physical equilibria between these compounds with the rest of the components present in the solution. Therefore, any compound that easily adsorbs to a particle or chemically interacts with another molecule will have greater difficulty crossing the membrane. 

It could be said that forward osmosis membrane is an excellent alternative for the retention and concentration of emerging pollutants present in urban wastewater, and therefore, allowing the elimination of this type of pollutant in the effluent avoids their effects imposing risks on health and environmental wellness.

#### 3.5.4. Ecological Risk of CECs after FO

Finally, considering the recovery obtained from the pollutants, the concentration of pollutant that could pass into clean water collected in the DS was evaluated. It was shown that for all pollutants and groups, the risk is low except for caffeine for Daphnia, which has a moderate risk. In this case, FO decreased its RQ from 32.30 (high risk) to 0.27 (moderate risk). Caffeine is found mainly in coffee, but it can be found to a lesser extent in other beverages such as those containing cocoa, chocolate, cola, tea, and some painkillers. Due to the large number of products that contain caffeine, it is the most popular psychoactive drug in the world. It is found in abundant concentrations in urban wastewater because part of it is not metabolized in the body [77]. There are numerous studies on the elimination of caffeine in wastewater, with membrane technology being a promising alternative with a high efficiency in its elimination [78]. In our case, the forward osmosis aquaporin membrane displays a 99% rejection rate of caffeine in pure water [41], so a similar rejection rate in urban wastewater is to be expected, which makes it an efficient disposal alternative.

## 4. Conclusions

The research carried out shows the importance of membrane fouling when urban wastewater is used and shows the need for the prior pretreatment of urban wastewater for this type of membrane. From the study of the different types of draw solutions of NaCl, MgCl_2_·6H_2_O, MgSO_4_·7H_2_O, CH_3_COONa, and C_6_H_12_O_6_, it was observed that NaCl allows higher permeate fluxes (10.6 LMH) and less reverse salt flux (0.73 GMH), which is why it was considered the most suitable salt. In addition, another advantage of using a 0.5 M salt concentration is that seawater could be used in coastal areas, since seawater has similar concentrations, which would reduce the cost of the process. A new criterion was tested to select adequate DS salts in terms of their diffusion coefficient and Vant’Hoff factors. The NaCl concentration of 0.5 M was chosen, as it is sufficient for the organic matter concentration process by FO and is more sustainable. Of the 51 pollutants studied, 18 frequently used in the daily life of the population were found in different concentrations in the urban wastewater of Valladolid. These pollutants are within the group of antibiotics (sulfamethoxazole, trimethoprim, sulfapyridine, ofloxacin, clarithromycin, levofloxacin, and ciprofloxacin) analgesics (naproxen, diclofenac, and ibuprofen) antihypertensives (atenolol), stimulants (caffeine), insect repellant (DEET), antiparasitics (fenbendazole), preservatives (methylparaben) and drugs in general for different diseases (gemfibrozil, atorvastatin, and carbamazepine). In all cases, good recovery was achieved, which increased with molecular weight, although chemical and electrostatic interactions also played a role. A clear trend of compound recovery with molecular weight was observed. Lower recoveries (<80%) were obtained with low-molecular-weight compounds such as ibuprofen, caffeine, and DEET, and higher recoveries (80–100%) with higher-molecular-weight compounds such as atorvastatin, ofloxacin, ciprofloxacin, fenbendazole, levofloxacin, and clarithromycin. The ecological risk of the contaminants in the aquatic ecosystem was evaluated, and it was demonstrated how the membrane allows their elimination or a significant reduction in their ecotoxicological risk through the concentration of these pollutants. The importance of osmotic washing should be highlighted in this type of system to recover the remnants that may have been adsorbed on the membrane.

## Figures and Tables

**Table 1:** List of the 51 contaminants studied from the urban WWTP of the city of Valladolid.

	Analytes		Analytes
1	Penicillin	27	Clarithromycin
2	Oxytetracycline	28	Erythromycin
3	Doxycycline	29	Naproxen
4	Tetracycline	30	Clofibrate
5	Marbofloxacin	31	Levofloxacin
6	Enrofloxacin	32	Norfloxacin
7	Danofloxacin	33	1,4-Benzoquinone
8	Sulfadiazine	34	Atorvastatin
9	Sulfathiazole	35	Atenolol
10	Sulfamethizole	36	Caffeine
11	Sulfadimidine	37	Atrazine
12	Sulfamethoxazole	38	N, N-diethyl-meta-toluamide (DEET)
13	Tylosin	9	Ciprofloxacin
14	Tiamulin	40	17-α-Ethynylestradiol
15	Apramycin	41	Crotamiton
16	Trimethoprim	42	Estrone
17	Florfenicol	43	Ethyl Paraben
18	Fenbendazole	44	Propyl Paraben
19	Dexametasone	45	Diclofenac Sodium Salt
20	Progesterone	46	Ibuprofen
21	Methyl paraben	47	Salicylic acid
22	Carbamazepine	48	Clofibric acid
23	Propanolol	49	Triclosan
24	Sulfapyridine	50	4-Hydroxybenzoic acid
25	Metronidazole	51	Gemfibrozil
26	Ofloxacin		

**Table 2:** Kinetic fouling constants.

	K (min^−1^)	$\mathrm{Average}\;J_{w}$ (L/m^2^h)	$\mathrm{Average}\;J_{s}$ (g/m^2^h)
Scenario A	0.010 ± 0.002	8.2 ± 0.7	0.67 ± 0.05
Scenario B	0.033 ± 0.002	6.6 ± 0.5	0.59 ± 0.05
Scenario C	0.111 ± 0.008	5.1 ± 0.4	0.75 ± 0.06

**Table 3:** Concentrations of contaminants found at the outlet of the desander of the urban WWTP from Valladolid.

Analytes	Concentration (ng/L)	Analytes	Concentration (ng/L)
Penicillin G	<MLD	**Naproxen**	1864.5
Oxytetracycline	<MLD	**Clarithromycin**	83.2
Doxycycline	<MLQ	Erythromycin	<MLQ
Tetracycline	<MLD	Clofibrate	<MLD
Marbofloxacin	<MLD	**Levofloxacin**	111.76
Enrofloxacin	<MLQ	Norfloxacin	<MLD
Danofloxacin	<MLD	1,4-Benzoquinone	<MLD
Sulfadiazine	<MLD	**Atorvastatin**	36.68
Sulfathiazole	<MLD	**Atenolol**	316.37
Sulfamethizole	<MLD	**Caffeine**	14,210.9
Sulfadimidine	<MLD	Atrazine	<MLQ
**Sulfamethoxazole**	218.05	**DEET**	72.44
Tylosin	<MLD	**Ciprofloxacin**	257.21
Tiamulin	<MLQ	17-α-Ethynylestradiol	<MLD
Apramycin	<MLD	Crotamiton	<MLD
**Trimethoprim**	93.54	Estrone	<MLD
Florfenicol	<MLD	Ethyl Paraben	<MLD
**Fenbendazole**	11.09	Propyl Paraben	<MLD
Dexametasone	<MLD	**Diclofenac Sodium Salt**	680.91
Progesterone	<MLD	**Ibuprofen**	5322.55
**Methyl paraben**	117.83	Salicylic acid	<MLD
**Carbamazepine**	28.76	Clofibric acid	<MLQ
Propanolol	<MLQ	Triclosan	<MLD
**Sulfapyridine**	11.05	4-Hydroxybenzoic acid	<MLD
Metronidazole	<MLD	**Gemfibrozil**	540.49
**Ofloxacin**	85.92		

**Table 4:** Ecological risk assessment of contaminants in the urban wastewater from the Valladolid WWTP.

ANALYTES	RQ Daphnia	RQ Fish	RQ Green Algae
Sulfamethoxazole	0.01	0.00	8.08
Diclofenac	0.03	0.02	0.05
Naproxen	0.01	0.01	0.02
Ibuprofen	0.59	0.13	1.33
Gemfibrozil	0.05	0.08	0.06
Atorvastatin	0.43	0.41	016
Ciprofloxacin	0.00	0.00	51.44
Ofloxacin	0.00	0.00	0.00
Carbamazepine	0.00	0.00	0.00
Caffeine	32.30	0.20	0.09
DEET	0.00	0.00	0.00
Fenbendazole	0.67	0.04	0.01
Methylparaben	0.00	0.00	0.00
Sulfapyridine	0.00	0.00	0.00
Levofloxacin	0.00	0.00	0.00
Clarithromycin	0.00	0.00	0.04
Atenolol	0.01	0.00	0.15
Trimethoprim	0.00	0.00	0.01

## Data Availability

Not applicable.

## Supplementary Materials

The following supporting information can be downloaded at: https://www.mdpi.com/article/10.3390/membranes12030293/s1, Table S1: Specifications for the Aquaporin Inside TM FO hollow fiber module as provided by the membrane manufacturer; Table S2: Operational conditions of the UHPLC-MS/MS equipment; Table S3: Method Limits of Detection (MLD) and Method Limits of Quantitation (MLQ); Table S4: Diffusion coefficient and Van’t Hoff values; Table S5: Ionic radius of the ions studied; Table S6: Chemical oxygen demand (COD) recovery in aquaporin forward osmosis membrane; Table S7: Inorganic carbon (IC) and Total Carbon (TC) recovery in aquaporin forward osmosis membrane; Table S8: Properties of compounds present in effluent of urban WWTP in the city of Valladolid; Table S9: Some properties of the compounds taken from the SciFinder database and additional information; Table S10: Further properties of the compounds taken from SciFinder database and additional information; Table S11: EC50 (mg/L) for daphnia, fish, and green algae for the 18 studied contaminants; Table S12: Evaluation of ecological risks of pollutants in the water recovered after passing through the membrane. References [47,48,49,50,52,68,69,70,71,72,73,74,75] are cited in the Supplementary Materials.
