# Supplementary material for: Ecological Risk Evaluation and Removal of Emerging Pollutants in Urban Wastewater by a Hollow Fiber Forward Osmosis Membrane
Source: Membranes (Basel). 2022 Mar 4;12(3):293. doi: 10.3390/membranes12030293 (PMC8949913; doi:10.3390/membranes12030293)
Supplement: Supplementary file 1 [file membranes-12-00293-s001.zip › membranes-1604665-supplementary.pdf]

Supplementary Materials

Ecological Risk Evaluation and Removal of Emerging Pollutants in Urban Wastewater by a Hollow Fiber forward Osmosis Membrane

Mónica Salamanca [1,2], Rebeca López-Serna [1,3], Laura Palacio [1,4,*], Antonio Hernandez [1,4], Pedro Prádanos [1,4] and Mar Peña [1,2]

[1] Institute of Sustainable Processes (ISP), University of Valladolid, Dr. Mergelina s/n, E-47011 Valladolid, Spain; monica.salamanca@uva.es (M.S.); rebeca.lopez@uva.es (R.L.-S.); antonio.hernandez@uva.es (A.H.); ppradanos@uva.es (P.P.); pena@iq.uva.es (M.P.)

[2] Department of Chemical Engineering and Environmental Technology, University of Valladolid, Dr. Mergelina s/n, E-47011 Valladolid, Spain

[3] Department of Analytical Chemistry, Faculty of Sciences, University of Valladolid Paseo Belén 7, E-47011 Valladolid, Spain

[4] Department of Applied Physics, Faculty of Sciences, University of Valladolid, Paseo Belén 7, E-47011, Valladolid, Spain

* Correspondence: laura.palacio@uva.es

Table S1. Specifications for the Aquaporin Inside TM FO hollow fiber module as provided by the membrane manufacturer.

Manufacturer	Aquaporin A/S (Copenhagen, Denmark)
Membrane module	HFFO.6
Active area	0.6 m^2
Number of fibers	8000
Fiber length	120 mm
Inner diameter of fibers	200 μm
Wall thickness of fiber	35 μm
Active layer	Polyamide thin film composite (TFC) with AQP vesicles
Porous support layer	Polysulfone (PS)
Cross sectional area shell	3.28E-05 m^2
Cross sectional area lumen	2.51E-04 m^2
Water flux (DI water vs.0.5M NaCl, 25 L/h as feed flow rate and 15 L/h as draw flow rate, temperature 298 K)	11 ± 1.5 L/m^2h
Specific reverse salt flux (DI water vs.0.5M NaCl, 25 L/h as feed flow rate and 15 L/h as draw flow rate, temperature 298 K)	0.15 ± 0.05 g/L

Table S2. Operational conditions of the UHPLC-MS/MS equipment.

Analytes	Q1	Q3	Declustering potential (V)	Collision energy (V)	Cell extraction potential (V)	Retention time (min)
Penicilina G	335	160	111	23	18	4.98
		176		19	16	
Oxytetracycline	461	426	151	27	30	3.64
		443		19	32	
Doxycycline	445	428	111	27	34	4.52
		410		37	36	
Tetracycline	445	410	51	27	32	3.30
		427		19	32	
Marbofloxacin	363	320	66	23	24	2.77
		327		25	24	
Enrofloxacin	360	316	91	29	38	4.18
		342		33	38	
Danofloxacin	358	340	86	33	24	4.16
		314		27	38	
Sulfadiazine	251	156	71	23	20	1.50
		108		31	12	
Sulfathiazole	256	156	96	21	8	1.84
		108		33	12	
Sulfamethizole	271	156	31	21	10	3.61
		108		35	12	
Sulfadimidine	279	186	86	25	22	3.76
		124		33	6	
Sulfamethoxazole	254	156	66	23	24	4.18
		108		33	16	
Tylosin	916	772	156	43	36	4.79
		174		51	10	
Tiamulin	494	192	51	29	10	4.72
		119		59	12	
Apramycin	271	156	50	20	19	3.57
		-		-	-	
Trimethoprim	291	230	51	33	18	2.18
		261		35	16	
Florfenicol	358	340	66	13	24	4.18
		241		25	14	
Fenbendazol	300	268	96	29	22	5.14
		159		49	10	
Dexamethasone	393	355	41	19	20	5.06
		147		39	10	
Progesterone	315	109	141	31	10	5.39
		297		23	28	
Methylparaben	153	121	66	21	14	4.48
		109		15	30	
Carbamazepine	237	194	66	29	12	4.94
		193		47	6	

Table S2. *Cont.*

Propanolol	260	183	66	25	12	4.47
		116		25	8	
Sulfapyridine	250	156	61	23	10	1.93
		108		35	12	
Metronidazole	172	128	41	21	6	0.91
		82		35	14	
Ofloxacin	362	318	86	29	26	5.91
		261		37	14	
Naproxen	231	185	56	21	12	5.15
		170		37	10	
Clarithromycin	748	590	96	25	40	6.19
		158		41	26	
Erythromycin	734	576	46	25	36	5.06
		158		37	22	
Clofibrate	243	169	86	17	22	5.38
		197		13	10	
Levofloxacin	362	318	6	27	20	3.90
		261		37	24	
Norfloxacin	320	276	96	27	18	3.93
		233		37	16	
1,4-Benzoquinone	109	81	121	19	4	5.09
		53		29	12	
Atorvastatin	559	440	26	33	38	5.32
		250		59	18	
Atenolol	267	145	11	33	24	0.80
		190		29	4	
Caffeine	195	138	71	27	18	3.81
		110		31	16	
Atrazine	216	174	71	25	22	5.02
		104		41	16	
DEET	192	119	56	23	10	5.25
		91		41	10	
Ciprofloxacin	332	314	31	29	38	4.07
		231		57	16	
17-α-Ethynylestradiol	297	107	71	33	12	5.56
		77		79	10	
Crotamiton	204	69	61	35	12	5.63
		136		27	14	
Estrone (E1)	271	253	101	19	10	5.54
		133		35	12	
Ethylparaben	165	137	-35	-20	-11	4.84
		136		-20	-13	
Propylparaben	179	137	-60	-20	-13	5.03
		136		-24	-17	
Diclofenac Sodium Salt	294	250	-10	-18	-7	5.47
		214		-28	-9	
Ibuprofen	2054	159	-35	-10	-15	5.38
		161		-12	-21	

Table S2. *Cont.*

Salicylic acid	137	93	-40	-22	-15	4.42
		-		-	-	
Clofibric acid	213	127	-35	-24	-15	5.14
		85		-14	-9	
Triclosan	287	287	-90	-6	-17	5.50
		142		-48	-15	
4-Hydroxybenzoic acid	137	93	-5	-16	-15	4.43
		65		-40	-13	
Gemfibrozil	249	121	-5	-30	-7	6.65
		127		-14	-5	

Table S3. Method Limits of Detection (MLD) and Method Limits of Quantitation (MLQ).

Analytes	MLD (ng/L)	MLQ (ng/L)	Analytes	MLD (ng/L)	MLQ (ng/L)
Penicillin G	218.14	727.13	Naproxen	8.88	29.60
Oxytetracycline	83.16	277.24	Clarithromycin	6.91	23.04
Doxycycline	30.63	214.30	Erythromycin	134.83	449.43
Tetracycline	7.62	25.41	Clofibrate	111.50	371.68
Marbofloxacin	160.94	536.46	Levofloxacin	7.16	23.86
Enrofloxacin	35.72	119.07	Norfloxacin	188.59	628.62
Danofloxacin	159.26	530.86	1,4-Benzoquinone	1371.98	4573.26
Sulfadiazine	93.97	313.24	Atorvastatin	0.84	2.81
Sulfathiazole	110.06	366.87	Atenolol	13.57	45.22
Sulfamethizole	31.04	103.48	Caffeine	4.41	14.71
Sulfadimidine	27.39	91.23	Atrazine	2.52	8.41
Sulfamethoxazole	2.45	8.17	DEET	0.82	2.72
Tylosin	104.45	348.17	Ciprofloxacin	5.05	16.85
Tiamulin	23.60	78.62	17-α-Ethynylestradiol	706.46	2354.87
Apramycin	125.33	417.76	Crotamiton	3.15	10.51
Trimethoprim	0.81	2.69	Estrone	300.91	1003.05
Florfenicol	502.39	1674.65	Ethyl Paraben	77.11	257.02
Fenbendazol	0.68	2.25	Propyl Paraben	138.19	460.64
Dexametasone	110.10	367.00	Diclofenac Sodium Salt	24.47	81.57
Progesterone	24.49	81.64	Ibuprofen	266.37	887.89
Methyl paraben	7.64	25.46	Salicylic acid	72.52	241.74
Carbamazepine	2.31	7.72	Clofibric acid	14.28	47.61
Propanolol	5.75	19.15	Triclosan	986.85	3289.51
Sulfapyridine	0.27	0.90	4-Hydroxybenzoic acid	112.73	375.76
Metronidazole	80.94	269.80	Gemfibrozil	5.27	17.56
Ofloxacin	6.23	20.75			

Table S4. Diffusion coefficient and Van´t Hoff values [1-4].

Salt	i theorist Van´t Hoff	i experimental Van´t Hoff	Diffusion coefficient (m^2/s)
NaCl	2	1.87	1.610
$MgCl_2$	3	2.84	1.249
$MgSO_4$	2	1.04	0.849
AcNa	2	1.92	1.200
Glucose	1	1.00	0.673

Table S5. Ionic radius of the ions studied [5].

Ion	Ionic radius (pm)
Ac^-	162
Cl^-	184
SO_4^{2-}	258
Na^+	102
Mg^{2+}	72

Table S6. Chemical Oxygen Demand (COD) recovery in aquaporin forward osmosis membrane.

Samples	COD recovery %
FS experiment	81.32
FS 1wash	27.75
FS 2wash	3.36
FS 3wash	10.08
total	122.51

Table S7. Inorganic Carbon (IC) and Total Carbon (TC) recovery in aquaporin forward osmosis membrane.

Samples	IC recovery %	TC recovery %
FS experiment	69.39	72.31
FS 1wash	9.15	10.02
FS 2wash	3.78	4.45
FS 3wash	1.82	2.07
total	84.15	88.84

Table S8. Properties of compounds present in effluent of urban WWTP in the city of Valladolid.

Analytes	MW (amu)	log K_{ow} at 25 °C	log D	Charge pH 7
Sulfamethoxazole	253.28	0.659	-0.558	Negative
Diclofenac	296.15	4.548	1.727	Negative
Naproxen	230.26	2.876	0.713	Negative
Ibuprofen	206.28	3.502	0.911	Negative
Gemfibrozil	250.33	4.302	2.050	Negative
Atorvastatin	558.64	3.846	1.135	Negative
Ciprofloxacin	331.34	1.625	1.625	Neutral
Ofloxacin	361.37	1.855	1.855	Neutral
Carbamazepine	236.27	1.895	1.895	Neutral
Caffeine	194.19	-0.628	-0.628	Neutral
DEET	191.27	2.419	2.419	Neutral
Fenbendazole	299.35	2.033	2.033	Neutral
Methylparaben	152.15	1.882	1.882	Neutral
Sulfapyridine	249.29	0.469	0.469	Neutral
Levofloxacin	361.37	1.855	1.855	Neutral
Clarithromycin	747.95	2.805	1.616	Positive
Atenolol	266.34	0.335	-2.097	Positive
Trimethoprim	290.32	0.594	0.273	Positive

Table S9. Some properties of the compounds taken from the SciFinder database and additional information.

**Analytes**	**Therapeutic subclass**	**Therapeutic class**	**MW (amu)**	**Molecular formula**	**Structure**	**pKa at 25 °C**
Ciprofloxacin	Fluroquinolones	Antibiotics	331.34	$C_{17}H_{18}FN_3O_3$	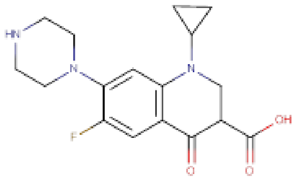	Most acidic: 6.43<br>Most basic: 8.68
Ofloxacin			361.37	$C_{18}H_{20}N_3FO_4$	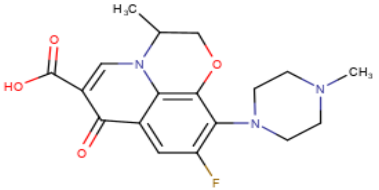	Most Acidic: 5.19<br>Most Basic: 7.37
Levofloxacin			361.37	C_{18} H_{20} F N_3 O_4	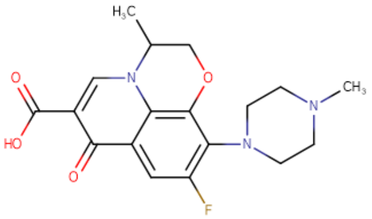	Most acidic: 5.19<br>Most basic: 7.37
Sulfamethoxazole	Sulfonamides		253.28	$C_{10}H_{11}N_3O_3S$	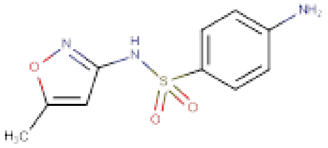	Most acidic: 5.81<br>Most basic: 1.39

Clarithromycin	Macrolides	747.95	$C_{38}H_{69}NO_{13}$	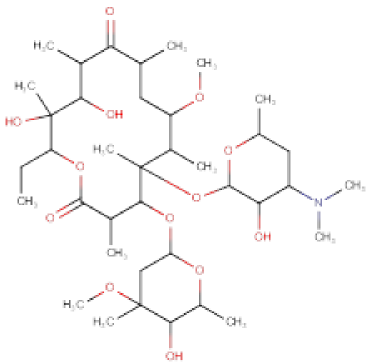	Most acidic: 13.08<br>Most basic: 8.16
Sulfapyridine	Antibacterial	249.29	$C_{11}H_{11}N_3O_2S$	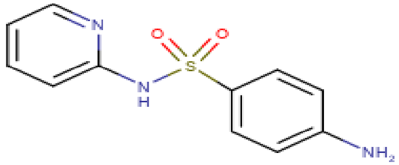	Most Acidic: 8.54<br>Most Basic: 2.13
Trimethoprim	Antibacterial	290.32	$C_{14}H_{18}N_4O_3$	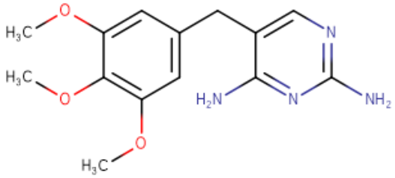	Most Basic: 7.04

Diclofenac			296.15	$C_{14}H_{11}Cl_2NO_2$	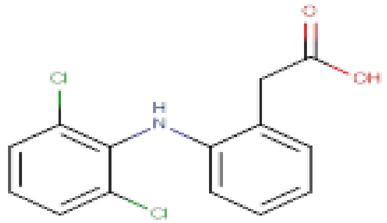	Most acidic: 4.18<br>Most basic: -2.26
Naproxen	AINE (antiinflamatorio no esteroideo)	Analgesics/anti-inflammatories	230.26	$C_{14}H_{14}O_3$	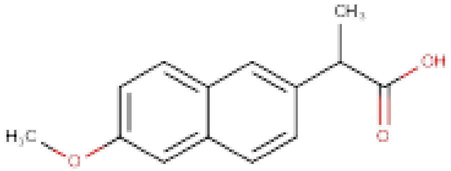	Most acidic: 4.84
Ibuprofen			206.28	$C_{13}H_{18}O_2$	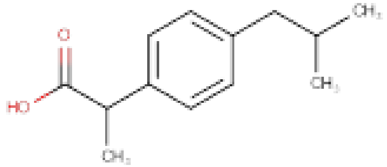	Most acidic: 4.41
Fenbendazole	Anti-parasitics		299.35	$C_{15}H_{13}N_3O_2S$	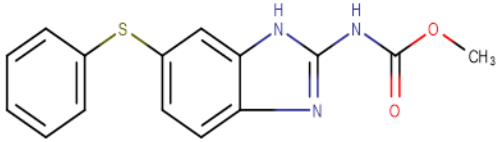	Most Acidic: 12.13

Methylparaben	Preservative		152.15	$C_8H_8O_3$	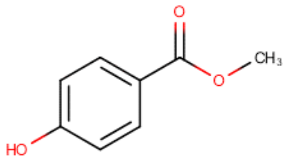	Most acidic: 8.31
Atorvastatin	Lipid-lowering drug	Statins	558.64	$C_{33}H_{35}FN_2O_5$	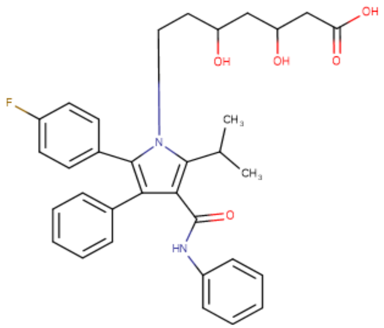	Most acidic: 4.29<br>Most basic: 0.38
Gemfibrozil		Fibric Acid Agents	250.33	$C_{15}H_{22}O_3$	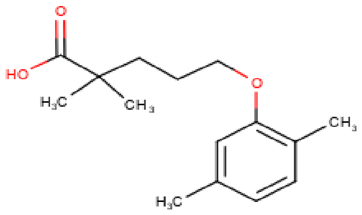	Most Acidic: 4.75
Carbamazepine		Psychiatric drugs/ anticonvulsants	236.27	$C_{15}H_{12}N_2O$	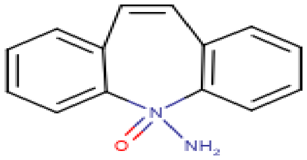	Most Acidic: 13.94<br>Most Basic: -0.49

Atenolol	Beta-Blockers	266.34	$C_4H_{22}N_2O_3$	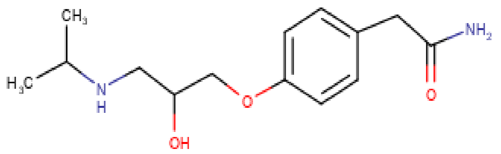	Most Acidic: 13.88<br>Most Basic: 9.43
Caffeine	Stimulant	194.19	$C_8H_{10}N_4O_2$	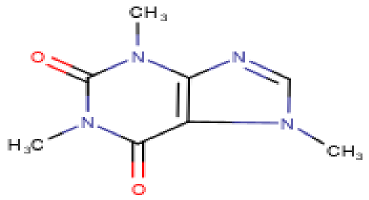	Most Basic: 0.52
DEET	Insect repellant	191.27	$C_{12}H_{17}NO$	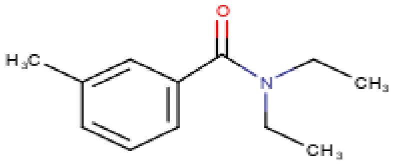	Most basic: -1.37

Table S10. Further properties of the compounds taken from SciFinder database and additional information.

Analytes	Boiling Temp. Tb (°C) at 101325 Pa	Vapor pressure Vp (Pa) at 25 °C	Supplier
Ciprofloxacin	581.80	2,99E-12	Sigma-Aldrich
Ofloxacin	571.50	6,70E-14	Fisher
Levofloxacin	571.50	8,93E-12	Sigma-Aldrich
Sulfamethoxazole	482.10	2,49E-07	Fisher
Clarithromycin	805.50	6,75E-28	Sigma-Aldrich
Sulfapyridine	473.50	3,90E-09	Fisher
Trimethoprim	526.00	3,74E-11	Sigma-Aldrich
Diclofenac	412.00	2,12E-05	Sigma-Aldrich
Naproxen	403.90	4,01E-05	Sigma-Aldrich
Ibuprofen	319.60	1,85E-02	Sigma-Aldrich
Fenbendazole	568.20	2,81E-15	Sigma-Aldrich
Methylparaben	265.50	7,40E-01	Sigma-Aldrich
Atorvastatin	722.20	9,12E-20	Sigma-Aldrich
Gemfibrozil	158.50	6,13E-07	Fisher
Carbamazepine	411.00	7,71E-05	Sigma-Aldrich
Atenolol	508.00	3,82E-11	Fisher
Caffeine	416.80	3,72E-07	Fisher
DEET	160.00	1,35E-03	Fisher

Table S11. EC50 (mg/L) for daphnia, fish and green algae for the 18 studied contaminants [6-13].

	EC50 (mg/L)		
**ANALYTES**	**Daphnia**	**Fish**	**Green algae**
Sulfamethoxazole	25.2	562.5	0.0
Diclofenac	22.0	38.4	14.5
Naproxen	126.1	190.0	96.6
Ibuprofen	9.0	42.0	4.0
Gemfibrozil	10.4	7.1	9.5
Atorvastatin	0.1	0.1	0.2
Ciprofloxacin	620.7	197.1	0.0
Ofloxacin	31.8	316.8	179.7
Carbamazepine	11.9	35.4	85.0
Caffeine	0.4	70.0	150.0
DEET	75.0	71.3	388.0
Fenbendazole	0.0	0.3	1.0
Methylparaben	24.6	160.0	31.4
Sulfapyridine	1.8	377.5	6.4
Levofloxacin	1790.0	19400.0	2440.0
Clarithromycin	25.7	280.0	2.1
Atenolol	51.4	774.3	2.1
Trimethoprim	123.0	635.0	16.0

Table S12. Evaluation of ecological risks of pollutants in the water recovered after passing through the membrane.

ANALYTES	RQ Daphnia	RQ Fish	RQ Green algae
Sulfamethoxazole	0.00	0.00	0.00
Diclofenac	0.00	0.00	0.00
Naproxen	0.00	0.00	0.00
Ibuprofen	0.00	0.02	0.00
Gemfibrozil	0.00	0.00	0.00
Atorvastatin	0.00	0.02	0.01
Ciprofloxacin	0.00	0.00	0.00
Ofloxacin	0.00	0.00	0.00
Carbamazepine	0.00	0.00	0.00
Caffeine	0.27	0.02	0.01
DEET	0.00	0.00	0.00
Fenbendazole	0.00	0.00	0.00
Methylparaben	0.00	0.00	0.00
Sulfapyridine	0.00	0.00	0.00
Levofloxacin	0.00	0.00	0.00
Clarithromycin	0.00	0.00	0.00
Atenolol	0.00	0.00	0.00
Trimethoprim	0.00	0.00	0.00

References

1. Robinson, R.A. and Stokes R.H. Electrolite solution, 2ª ED, UK, Dover, 2002.
2. Lide, D.R, CRC Handbook of Chemistry and Physics, Special Student Edition, 75th ed., CRC Press, Boca Raton, USA, 1994.
3. Gilbert, L. The Osmotic Pressure of Concentrated Solutions and the Laws of the Perfect Solution. *Journal of the American Chemical Society* **1908**, *30 (5)*, 668–683. https://doi.org/10.1021/ja01947a002
4. Castaldi, M., D´Errico, G., Paduano, L., Vitalgliano, V. Transport Properties of the Binary System Glucose−Water at 25 °C. A Velocity Correlation Study. *J. Chem. Eng. Data* **1998**, *43*, 653-657. https://doi.org/10.1021/je980054c
5. Wolfram Research, Inc. Wolfram|Alpha Notebook Edition, Champaign, IL, 2020.
6. López-Serna, R.; Postigo, C.; Blanco, J.; Pérez, S.; Ginebreda, A.; de Alda, M.L.; Petrović, M.; Munné, A.; Barceló, D. Assessing the Effects of Tertiary Treated Wastewater Reuse on the Presence Emerging Contaminants in a Mediterranean River (Llobregat, NE Spain). *Environmental Science and Pollution Research* **2012**, *19*, 1000–1012, https://doi.org/10.1007/s11356-011-0596-z.
7. Reis, E.O.; Santos, L.V.S.; Lange, L.C. Prioritization and Environmental Risk Assessment of Pharmaceuticals Mixtures from Brazilian Surface Waters. *Environmental Pollution* **2021**, *288*, https://doi.org/10.1016/j.envpol.2021.117803.
8. Dai, G.; Wang, B.; Fu, C.; Dong, R.; Huang, J.; Deng, S.; Wang, Y.; Yu, G. Pharmaceuticals and Personal Care Products (PPCPs) in Urban and Suburban Rivers of Beijing, China: Occurrence, Source Apportionment and Potential Ecological Risk. *Environmental Science: Processes and Impacts* **2016**, *18*, 445–455, https://doi.org/10.1039/c6em00018e.
9. Jin, S.O.; Park, J.; Jung Lee, M.; Young Park, S.; Lee, J.; Choi, K. Hazard/Risk Assessment. Ecological hazard assessment of major veterinary benzimidazoles: acute and chronic toxicities to aquatic microbes and invertebrates. *Environmental Toxicology and Chemistry* **2006**, *25 (8)*, 2221-6.
10. Wagil, M.; Białk-Bielińska, A.; Puckowski, A.; Wychodnik, K.; Maszkowska, J.; Mulkiewicz, E.; Kumirska, J.; Stepnowski, P.; Stolte, S. Toxicity of Anthelmintic Drugs (Fenbendazole and Flubendazole) to Aquatic Organisms. *Environmental Science and Pollution Research* **2015**, *22*, 2566–2573, https://doi.org/10.1007/s11356-014-3497-0.
11. Cui, J.; Fu, L.; Tang, B.; Bin, L.; Li, P.; Huang, S.; Fu, F. Occurrence, Ecotoxicological Risks of Sulfonamides and Their Acetylated Metabolites in the Typical Wastewater Treatment Plants and Receiving Rivers at the Pearl River Delta. *Science of the Total Environment* **2020**, *709*, https://doi.org/10.1016/j.scitotenv.2019.136192.
12. Park, J.; An, S.; Jho, E.H.; Bae, S.; Choi, Y.; Choe, J.K. Exploring Reductive Degradation of Fluorinated Pharmaceuticals Using Al2O3-Supported Pt-Group Metallic Catalysts: Catalytic Reactivity, Reaction Pathways, and Toxicity Assessment. *Water Research* **2020**, *185*, https://doi.org/10.1016/j.watres.2020.116242.
13. Visca Palermo, M. Emerging Pollutants Removal in Wastewater Treatment Plants: A review and their implications in a river basin in Uruguay. This research is done for the partial fulfilment of requirements for the Master of Science degree at the UNESCO-IHE Institute for Water Education, Delft, the Netherlands. 2017. https://saniup.org/wp-content/uploads/2018/01/UWS-SE-CALI-2017-17-Mercedes-Visca-Palermo_EXAM-VERSION.pdf
